# Supplementary material for: Amelioration of Functional, Metabolic, and Morphological Deterioration in the Retina following Retinal Detachment by Green Tea Extract
Source: Antioxidants (Basel). 2024 Feb 15;13(2):235. doi: 10.3390/antiox13020235 (PMC10886023; doi:10.3390/antiox13020235)
Supplement: Supplementary file 1 [file antioxidants-13-00235-s001.zip › antioxidants-2839451-supplementary.pdf]

Supplementation

Figure S1. Diagram shows representative multiplex blotting of cytokines from Non-RD, RD, and GTE (142.9 mg/kg) group.

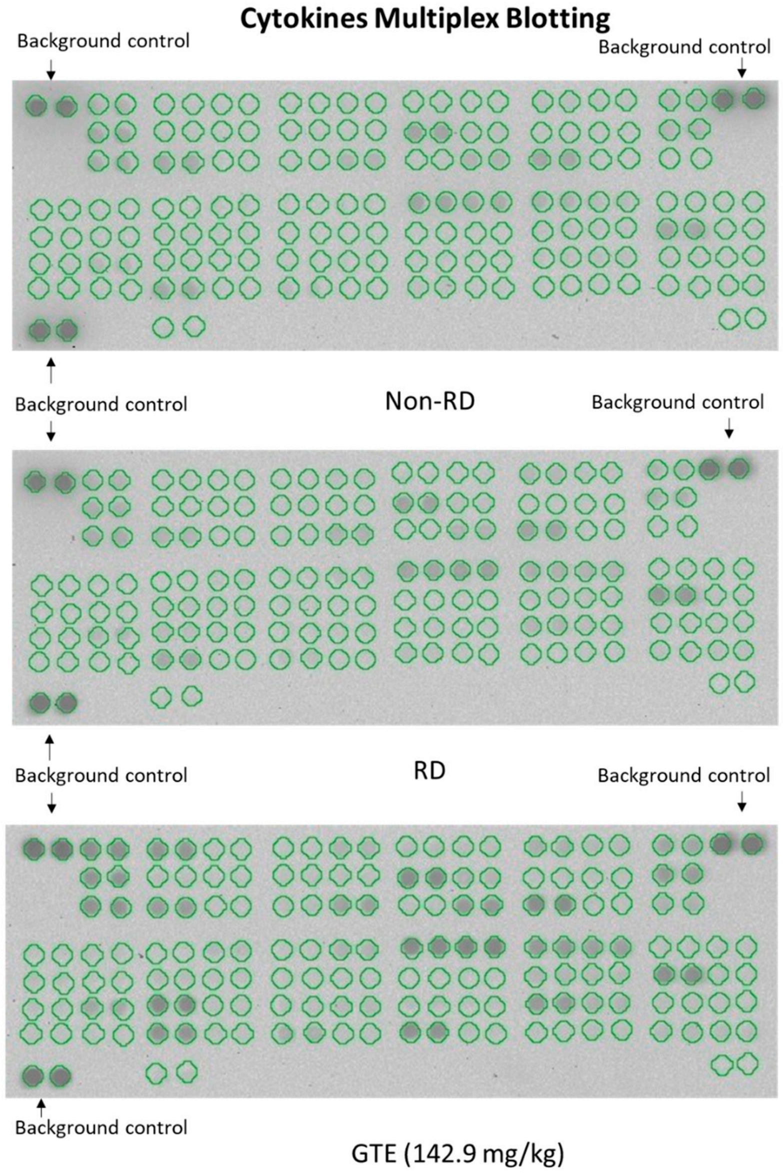

Table S1. Results of normalized ONL thickness from different groups, Non-RD, RD, EGCG 96.25mg/kg, EGCG 192.5mg/kg, and GTE 142.5mg/kg in batch 1 analysis as shown in Figure 3.

**Batch 1**					
	**Normalized Thickness**				
	Non-RD	RD	EGCG 96.25mg/kg (T1)	EGCG 192.5mg/kg (T2)	GTE 142.5mg/kg (GTE1)
	0.97	1.29	1.13	0.96	0.98
	0.94	1.27	0.91	1.20	1.19
	0.80	1.04	1.54	0.97	0.94
	1.11	1.21	0.92	0.81	
	0.89	1.00	1.30	0.76	0.80
	1.07	1.00	1.03	0.70	0.81
	0.89	1.24	1.00	0.98	0.92
	0.90	0.71	1.53	1.43	0.97
	1.15	1.03	0.56	0.91	0.91
	1.01	1.23	1.18	1.02	0.86
	0.82	1.28	0.86	1.10	
	1.14	0.97	0.88	0.78	0.86
	0.97	0.73	0.80	1.09	1.21
	0.92	0.85	0.96	1.44	0.84
	0.95	0.70	0.75		1.08
	0.91	0.94	0.65	0.95	0.80
	0.81	0.51	0.54	0.93	0.87
	0.76	0.42	0.85	1.35	0.85
	1.10	0.70	0.91	0.79	0.84
	0.74	1.23	1.16	0.75	1.09
	1.09	0.64	0.91	1.18	0.97
	0.88	1.14	0.82	1.01	0.86
	1.01	1.17	1.39	0.93	1.20
	0.94		1.26	1.03	0.69
		1.49		0.68	0.86
	0.90	1.91	1.56	0.93	
	1.10	1.16	1.30	0.64	0.96
	0.59	2.77	0.73	1.26	0.95
	0.97	2.10	1.96	1.41	0.99
	0.99	0.94	1.31	0.93	0.73
**Mean**	0.94	1.13	1.06	1.00	0.93
SD	0.13	0.48	0.33	0.23	0.13
SEM	0.02	0.09	0.06	0.04	0.02
T-test: Non-RD-RD	0.05				
T-test: T1-RD			0.54		
T-test: T2-RD				0.20	
T-test: GTE1-RD					0.04

Table S2. Results of normalized ONL thickness from different groups, Non-RD, RD, GTE 275 mg/kg, and EGCG 500 mg/kg in batch 2 analysis as shown in Figure 3.

**Batch 2**				
	Normalized Thickness			
	Non-RD	RD	GTE 275mg/kg (GTE2)	GTE 550mg/kg (GTE3)
	0.90		1.22	1.14
	1.22	1.51	1.43	0.80
	1.08	1.64	1.14	0.75
	0.94	1.80	1.41	0.72
	0.96	1.69	1.25	0.64
	1.08	1.82	1.48	0.80
	1.08	2.18	0.84	0.82
	0.87	1.45	1.49	0.55
	1.23	2.15	1.58	0.78
	0.91	2.49	1.31	0.67
	0.98	1.04	1.04	1.31
	1.18	1.35	1.22	0.94
	1.07	1.60	1.38	0.94
	0.71	1.25	1.17	0.67
	1.17	0.75	1.34	0.84
	0.90	0.98	1.34	0.83
	1.20	1.34	1.56	0.80
	0.98	1.13	1.32	0.91
	0.99	1.03	1.24	0.86
	0.94		1.06	0.79
	0.82	1.19	0.97	0.59
	0.65	0.87	1.13	0.74
	0.69	1.09	0.90	1.36
	0.82	1.08	0.86	0.85
	0.82	0.94	0.94	0.98
	1.10	0.97	1.04	0.94
	1.46	0.95	1.12	0.84
	1.01	1.00	1.23	1.87
	0.78	1.00	1.04	0.72
	1.22	0.84	0.88	0.82
**Mean**	0.99	1.33	1.20	0.88
SD	0.19	0.45	0.21	0.26
SEM	0.03	0.08	0.04	0.05
T-test: Non-RD-RD	0.0008			
T-test: GTE2-RD			0.18	
T-test: GTE3-RD				0.0000

Table S3. Scotopic (a) and phototopic (b) results of ERG of Non-RD, RD, EGCG 96.25mg/kg, EGCG 192.5mg/kg, and GTE 142.5mg/kg in batch 1 analysis as shown in Figure 4.

(a)

**Batch 1**										
Scotopic										
	**1.0 (P)cd.s/m^2**					**1.0 (P)cd.s/m^2**				
	**a-wave amplitude**					**b-wave amplitude**				
	Non-RD	RD	T1	T3	GTE1	Non-RD	RD	T1	T3	GTE1
Right Eye1	156.5	84.17	71	99.93	123.5	522.6	403.8	238.8	333.2	336.8
	149.9	120.6	79.75	105.2	96.12	500.9	403.9	228.5	299.6	319.2
	135.1	118.2	56.83	97.72	87.66	524	403.9	223.5	276.8	278
	163	133.4	76.37	90.25	109.7	512.4	432.6	237	293.3	352.2
	150.5	111.9	57.17	112.3	99.3	501.3	402.7	238.6	297.4	324.1
	147.8	113.7	67.13	99.61	103	485.6	406.6	229.4	293.3	317.4
Repeat	140.5					510.1				
	132.1					504.3				
	140.1					513.9				
	141					495				
Left Eye1	186.1	69.69	44.89	88.69	96.55	682.3	303	157.8	294.8	306
	181	77.81	68.05	100.1	88.72	672.1	288.2	159.3	271.8	296.5
	183.3	103	38.28	90.25	85.04	722.8	301.5	150.9	261.8	265.8
	195.8	103.5	58.6	72.96	83.47	709	338.3	156.3	252.5	292.2
	164.7	79.98	52.7	92.05	94.38	601	254.1	150.2	284.4	314.1
	164.5	80.32	51.56	87.16	88.53	607.8	293.2	150.7	268	284.5
Repeat	140.3					590				
	145					612.7				
	156.3					620.1				
	165.2					637.7				
Right Eye2	53.28	65.29	43.73	107.4	113.6	231.3	193.8	149.2	188.7	305.5
	56.1	73.27	56.91	63.89	102.7	273.7	225.4	169.2	158.4	304.1
	56.68	49.49	50.56	26.43	93.47	274.3	201.9	153.3	149.4	289
	59.14	85.18	49.54	89.44	119.8	255.2	217	155.3	212.4	274.6
	72.74	66.09	49.63	74.61	99.61	284.9	221	150	191.3	285.9
	58.37	65.59	49.25	69.44	103.8	256.5	208.5	149.6	173.8	281.7
Left Eye2	78.55	46.66	53.29	64.27	134	350.3	74.16	233.9	77.83	409.5
	92.62	32.88	79.64	18.86	119	348.9	124.3	256.8	40.8	409
	75.08	16.72	54.28	6.287	103.3	363.2	89.53	229	51.7	379.3
	89.3	24.05	50.76	55.09	124	365	99.54	236.4	115	392.7
	99.25	24.66	58.06	30.21	134.8	389.5	117.2	236.7	42.25	403.5
	89.58	22.71	57.51	26.31	121.3	360.8	100.9	233.6	44.67	395.2
Right Eye3	123.5	63.96	62.89	23.74	114.2	325.9	219.7	164.70	43	324.7
	88.09	66.69	49.6	0	118.4	269.3	220.9	168.8	30.89	333
	90.45	56.01	60.22	14.53	105.8	255.5	216.1	165.2	44.98	312.6
	64.14	54.07	63.39	31.58	93.48	248.3	207	157.6	52.77	328
	77.74	55.17	46.96	43.58	105.1	253.4	201.9	161.6	61.88	283.3
Left Eye3	114.1	73.84	86.87	0	115.8	372.5	246	246.8	40.39	321.5
	119.3	68.49	77.66	11.94	104.3	344.4	240.7	252.1	30.66	316.4
	99.08	73.34	89.96	21.31	114.7	337.8	239.2	2424	22.98	298.5
	76.31	80.88	78.19	19.11	67.13	313.6	266.8	220.4	46.4	282
	90.53	84.44	96.49	0	118	333.6	253.8	245.3	25.87	289.2
Mean	118.16	71.93	61.40	56.89	105.36	436.51	247.56	261.19	155.09	320.76
SD	42.09	29.17	14.37	37.81	15.16	149.39	98.61	384.31	110.87	41.50
REM	6.34	4.86	2.40	6.30	2.53	22.52	16.44	64.05	18.48	6.92
t-test compare to RD =	0.000		0.065	0.071	0.000	0.000		0.842	0.001	0.000

(b)

Batch 1					
Phototopic					
	4-LA 3.0 ERG				
	b-wave amplitube				
	Neg R1	Pos R1	T1 R1	T3 R1	GTE R1
Right Eye1	106.8	86.77	50.97	39.98	74.12
Left Eye1	140.1	39.41	36.05	51.3	77.51
Right Eye2	76.08	41.02	43.18	42.17	67.37
Left Eye2	86.3	27.27	56.77	19.71	94.53
Right Eye3	34.27	32.87	21.34		74.12
Left Eye3	52.17	64.58	37.45		77.51
Mean	82.62	48.65	40.96	38.29	77.53
SD	37.973	22.612	12.462	13.321	9.116
REM	5.725	3.769	2.077	2.220	1.519
t-test compare to RD =	0.096	0.096	0.487	0.389	0.025

Table S4. Scotopic (a) and phototopic (b) results of ERG of Non-RD, RD, EGCG 96.25mg/kg, EGCG 275 mg/kg, and GTE 550 mg/kg in batch 2 analysis as shown in Figure 4.

(a)

Batch 2									
Scotopic									
	1.0 (P)cd.s/m²					1.0 (P)cd.s/m²			
	a-wave amplitude					b-wave amplitude			
	Non-RD	RD	GTE2	GTE3		Non-RD	RD	GTE2	GTE3
Right Eye1	70.2	130.9	83.36	65.64		255.7	305.5	301	204.8
	80.18	118.5	89.69	75.3		235.6	305.2	325.2	165.7
	61.16	108.4	97.56	59.75		233.7	279.3	335.3	183.1
	97.91	91.75	95.95	77.12		264.1	277.2	350.6	234.2
	43.16	116.4	88.12	63.78		233.2	320.5	312.1	202
Left Eye1	70.6	118	75.72	71.55		300.6	355.1	206.8	210.8
	66.96	121.2	72.11	76.38		293.3	347.3	248.4	180.2
	104.6	115	104.8	63.92		323.5	349.6	285.3	187.9
	129.4	122.7	74.44	77.12		358.4	350.9	266.9	227.7
	70.23	117.4	85.22	65.48		289.7	364.2	246.9	198.8
Right Eye2	155.4		57.62	43.94		504.6		153.3	61.86
	127.2		51.3	48.81		470.9		150.5	80.09
	146.9		39.9	30.35		486.1		130.8	81.61
	136.5		32.89	54.81		452.8		130.6	67.05
	135.6		56.27	37.68		439.3		158.5	99.44
Left Eye2	202.8		109.9	54.23		749.4		248.3	94.94
	160.2		127.5	49.02		695.7		282.5	92.14
	192.9		69.41	35.63		693.3		231.5	98
	190.8		76.47	66.65		692.1		246	86.64
	171.3		54.88	42.75		664.6		243.1	111.8
Right Eye3	122.7	46.44	93.12	56.98		292.6	160.6	172.9	183
	110.6	50.94	70.08	73.93		268.9	150.7	116	205.5
	105.5	60.89	67.75	71.09		245	179.5	125.3	204.9
	98.88	33.6	67.02	57.52		282.1	155.1	128	205.3
	94.32	45.73	66.72			259.6	120	127.4	191.4
Left Eye3	118	68.59	69.17	80.23		374.6	265.2	128.5	249.6
	127.7	70.8	48.76	100.2		363.7	242.5	83.4	280.7
	114.1	68.02	42.74	81.76		342.9	263.4	100.4	263.7
	123.6	35.95	37.69	80.19		368.2	250.5	98.06	265.3
	123.2	73.85	38.08	83.68		350.6	242.1	127.9	267.3
Mean	118.42	85.753	71.47467	63.63759		392.8267	264.22	202.0487	172.849
SD	40.43258	33.45784	23.28424	16.48181		159.1891	76.62837	81.73757	67.77415
REM	7.38	7.48	4.25	3.01		29.06	17.13	14.92	12.37
t-test compare to RD =	0.003237		0.107092	0.011185		0.000421		0.009022	0.00011

(b)

Batch 2				
Phototopic				
	4-LA 3.0 ERG			
	b-wave amplitube			
	Non-RD	RD	GTE2	GTE3
Right Eye1	57.20	74.76	45.33	50.25
Left Eye1	65.72	111.50	36.09	54.84
Right Eye2	119.50		30.12	64.23
Left Eye2	187.30		64.52	60.40
Right Eye3	65.06	55.45	35.00	45.71
Left Eye3	94.25	72.88	25.82	51.28
Mean	98.17	78.65	39.48	54.45
SD	49.48	23.56	13.91	6.86
REM	20.20	11.78	5.68	2.80
t-test compare to RD =	0.43		0.04	0.13

Table S5. Summary of the results of (a) caspase-3, (b) sphingomyelin, (c) protein carbonyl, and (d) HIF-1α levels in the retina in non-RD, RD, and GTE (142.9 mg/kg) group for Figure 6.

(a)

	Conc of Caspase 3 /protein (ng/mg)		
	Non-RD	RD	GTE
	1.904	1.969	
	1.368	2.912	1.726
	0.529	2.527	2.170
	2.690	1.960	2.402
	0.687	2.594	2.446
	0.566	2.577	2.573
Mean	1.291	2.423	2.263
SD	0.873	0.380	0.334
SEM	0.356	0.155	0.149
ANOVA (p =)	0.012		

(b)

	Conc. of Sphingomyelin/protein (nmol/μg)		
	Non-RD	RD	GTE
	0.029	0.015	0.014
	0.032	0.008	0.006
	0.021	0.008	0.010
	0.017	0.007	0.009
	0.023	0.029	0.010
	0.026	0.031	0.003
Mean	0.025	0.016	0.009
SD	0.006	0.011	0.004
SEM	0.002	0.004	0.002
ANOVA (p =)			0.007

(c)

	Conc. of protein carbonyl/protein (nmol/mg)		
	Non-RD	RD	GTE
	0.056	0.129	0.013
	0.035	0.056	0.024
		0.042	0.015
	0.093	0.097	0.011
	0.037	0.050	0.015
	0.070	0.048	0.019
Mean	0.058	0.070	0.016
SD	0.024	0.035	0.005
SEM	0.011	0.014	0.002
ANOVA (p =)			0.005

(d)

	Conc. of HIF-1a in each treatment (pg/µg)		
	Non-RD	RD	GTE
	0.055	0.164	0.058
	0.049	0.119	0.042
	0.040	0.105	0.083
	0.092	0.079	0.044
	0.063	0.088	0.080
	0.053	0.128	0.071
Mean	0.059	0.114	0.063
SD	0.018	0.031	0.018
SEM	0.007	0.013	0.007
ANOVA (p =)		0.001	

Table S6. Summary of results of differential expression of cytokines in the vitreous humors of different study groups. The expression of cytokines in the non-RD group was normalized as 1 whereas the fold changes in cytokine expressions in the other groups were compared. ANOVA with post hoc Tukey and Dunnett's test and t-test were used for the comparison where appropriate.

	Normalized Resonse	Fold change reponse												
Analyte	Mean Non-RD	Mean RD	Mean GTE	SD Non-RD	SD RD	SD GTE	SEM Non-RD	SEM RD	SEM GTE	Sig. diff. gp (Non-RD, RD, GTE)	ANOVA (p-value)	t-test Non-RD vs RD	t-test RD vs GTE	Expression
CCL2/JE/MCP-1	1	18.47	3.89	0.20	0.61	0.37	0.12	0.35	0.21	RD	0.010			+
CCL5/RANTES	1	0.36	0.89	0.20	0.59	0.48	0.12	0.34	0.28	RD	0.032			-
CCL11/Eotaxin	1	0.53	0.58	0.11	0.15	0.36	0.07	0.09	0.21	Non-RD	0.002			+
CCL17/TARC	1	0.84	1.51	0.18	0.25	0.16	0.11	0.15	0.09	GTE	0.004			+
CCL20/MIP-3α	1	38.70	21.38	0.17	0.61	1.00	0.10	0.35	0.58	Non-RD	0.050			-
CCL22/MDC	1	0.52	0.74	0.12	0.24	0.13	0.07	0.14	0.08	RD	0.001			-
CX3CL1/Fractalkine	1	0.56	1.00	0.26	0.21	0.16	0.15	0.12	0.09	RD	0.013			-
EG-VEGF/PK1	1	0.62	1.08	0.20	0.33	0.32	0.11	0.19	0.19	RD		0.035		-
Galectin-1	1	1.92	1.22	0.17	0.31	0.18	0.10	0.18	0.10	RD	0.019			+
GDF-15	1	4.52	3.15	0.06	0.39	0.09	0.03	0.22	0.05	Non-RD	0.003			-
ICAM-1/CD54	1	0.50	1.02	0.07	0.41	0.42	0.04	0.24	0.24	RD	0.046			-
IL-1α/IL-1F1	1	0.77	4.20	0.11	0.16	1.62	0.06	0.09	0.93	RD		0.033		-
IL-2	1	0.64	0.93	0.17	0.22	0.21	0.10	0.13	0.12	RD		0.020		-
IL-4	1	0.46	1.20	0.14	0.49	0.83	0.08	0.28	0.48	RD		0.010		-
IL-6	1	0.63	0.69	0.23	0.16	0.44	0.13	0.09	0.25	RD		0.040		-
IL-13	1	0.58	3.56	0.14	0.36	1.56	0.08	0.21	0.90	RD		0.020		-
IL-17A	1	1.18	1.95	0.27	0.22	0.24	0.16	0.13	0.14	GTE	0.009			+
LIF	1	0.67	0.75	0.08	0.19	0.44	0.05	0.11	0.25	Non-RD		0.007		+
Lipocalin-2/NGAL	1	1.89	2.01	0.14	0.21	0.44	0.08	0.12	0.25	Non-RD		0.017		-
Osteopontin (OPN)	1	5.99	3.14	0.22	0.62	0.30	0.13	0.36	0.17	Non-RD	0.033			-
Prolactin	1	0.03	0.06	1.05	0.17	0.31	0.61	0.10	0.18	RD			0.050	-
TWEAK/TNFSF12	1	0.53	0.97	0.08	0.08	0.16	0.04	0.04	0.09	RD	0.000			-
Neprilysin/CD10	1	0.63	0.96	0.22	0.06	0.22	0.12	0.03	0.13	RD	0.029			-
Clusterin	1	1.58	2.03	0.16	0.15	0.08	0.09	0.09	0.05	Non-RD	0.000			-
CNTF	1	1.50	0.80	0.13	0.35	0.18	0.07	0.20	0.10	RD	0.032			+
Fibulin 3	1	2.32	1.03	0.17	0.23	0.22	0.10	0.13	0.13	RD	0.001			+
HGF	1	0.50	1.82	0.11	0.51	0.70	0.07	0.30	0.40	RD		0.021		-
MAG/Siglec-4a	1	0.65	1.03	0.13	0.28	0.24	0.07	0.16	0.14	RD	0.040			-
MMP-3	1	15.93	9.99	0.23	0.16	0.30	0.13	0.09	0.17	Non-RD	0.000			-
Osteoprotegerin/TNFRSF11B	1	0.69	0.98	0.18	0.12	0.24	0.10	0.07	0.14	RD		0.032		-
SCF	1	0.61	0.88	0.03	0.34	0.27	0.02	0.20	0.15	RD	0.035			-
Serpin E1/PAI-1	1	34.54	3.74	0.14	0.72	0.49	0.08	0.42	0.28	RD	0.017			+
Adiponectin/Acrp30	1	4.63	1.37	0.14	0.34	0.38	0.08	0.20	0.22	RD	0.001			+
DPPIV/CD26	1	3.13	0.87	0.34	0.49	0.21	0.19	0.28	0.12	RD	0.011			+
Fetuin A/AHSG	1	0.87	0.67	0.56	0.04	0.05	0.32	0.03	0.03	GTE			0.000	-
FGF-21	1	0.59	0.73	0.07	0.29	0.33	0.04	0.17	0.19	Non-RD	0.024			+
IGF-I	1	16.35	2.51	0.14	0.69	0.28	0.08	0.40	0.16	RD	0.016			+
IGFBP-3	1	5.22	1.84	0.25	0.35	0.33	0.14	0.20	0.19	RD	0.001			+
